# Effect of Vacuum Heat Treatment on the Element Diffusion Behavior and Corrosion Resistance of Al_2_O_3_-3wt.%TiO_2_ Coating of Q235 Steel

**DOI:** 10.3390/ma15030848

**Published:** 2022-01-23

**Authors:** Yulin Ma, Guang Liu, Xinyu Wang, Xupeng Zhang, Jun Zhang, Jun Cheng

**Affiliations:** 1 Key Laborotary of Research and Application of Multiple Hard Films, Shenyang University, Shenyang 110044, China; LiuGuang_819@163.com (G.L.); wxy9942@163.com (X.W.); zxpdkdkd@163.com (X.Z.); zhjun88@126.com (J.Z.); 2 Northwest Institute for Nonferrous Metal Research, Shaanxi Key Laboratory of Biomedical Metal Materials, Xi’an 710016, China; 3 State Key Laboratory of Powder Metallurgy, Central South University, Changsha 410083, China

**Keywords:** vacuum heat treatment, Al_2_O_3_-3wt.%TiO_2_, plasma spraying, Rockwell hardness, corrosion resistance

## Abstract

In this study, we address the effect of vacuum heat treatment on the morphology of Al_2_O_3_-3wt.%TiO_2_ coating, element diffusion behavior, coating hardness, and corrosion resistance. The pores, cracks, and non-liquefied particles on the as-heat treated coating surface of the vacuum-heat-treated coating were observed and compared with the as-sprayed coating using a scanning electron microscope. The diffusion behavior of the elements in the coating was demonstrated by using a line scanning of a cross-section of the coating. Hardness and corrosion-resistance test results were used to judge the effect of a vacuum heat treatment on the coating. The research results show that compared with atmospheric heat treatment, the vacuum heat treatment had less effect on the pores, cracks, and non-liquefied particles on the surface of the coating. However, in the absence of new oxide formation, the pores and cracks in the cross-section of the coating were significantly improved by the vacuum heat treatment. The surface hardness and corrosion resistance of the coating were significantly improved. The crack defects were eliminated, and the uniformity of TiO_2_ distribution was improved, which are the main factors that improved the coating performance after vacuum heat treatment. The combination of the coating and the substrate is strengthened, and an Al_2_O_3_ and TiO_2_ interdiffusion zone is formed when the coating undergoes vacuum heat treatment, which is the main mechanism improving the performance of the AT3 coating.

## 1. Introduction

In order to further improve the corrosion resistance and wear resistance of metal parts, ceramic powder materials are widely used in plasma spraying to prepare metal surface coatings [1,2,3,4,5,6]. Al_2_O_3_-*x*wt.%TiO_2_, as one of the most widely used ceramic coating materials, is used in plasma spraying to improve the corrosion resistance of metal surfaces [7,8]. Compared with a single alumina coating, an Al_2_O_3_-*x*wt.%TiO_2_ coating doped with TiO_2_ has better wear resistance and toughness, despite the loss of some surface hardness [9,10,11]. Nevertheless, the pores, cracks, and non-liquefied particles in the Al_2_O_3_-*x*wt.%TiO_2_ coating prepared by plasma spraying are the main factors affecting the performance of the coating [12,13,14]. At present, there is much research on ceramic coatings doped with TiO_2_ in alumina, and the effect of TiO_2_ content on coating performance has become a research hotspot [15,16]. Recent research has reported that adding TiO_2_ to Al_2_O_3_ increased its corrosion resistance [17,18]. However, with the increase in TiO_2_ content in the powder, the thermal insulation property of coatings decreases [19]. At present, there are many studies on plasma coatings of Al_2_O_3_-13wt.%TiO_2_ and Al_2_O_3_-3wt.%TiO_2_ [16,20,21,22].

Studies have found that the performance of ceramic coatings can be significantly improved by heat treatment [23]. Li [24] studied the effect of heat treatment on the corrosion and wear resistance of Ni-based coatings in simulated seawater. The corrosion resistance and wear resistance of the coating were significantly improved, which can be attributed to the decrease in the number of pores and cracks on the surface caused by oxidation during heat treatment. Liu [25] studied the effect of heat treatment at 350–650 °C on the performance of Fe-based amorphous coatings. The corrosion resistance of the amorphous coating after heat treatment was significantly reduced. With the increase in heat-treatment temperature, the presence of oxidation and cracks on the surface increased significantly, which was the main reason for the degradation in performance. Zhao’s research pointed out that 800 °C heat treatment significantly improved the corrosion resistance of TiN coatings [26]. TiO_2_ and Ti_3_O are formed in the coating because oxygen participates in the reaction during heat treatment, reducing porosity and cracks in the coating.

Heat treatment also significantly improves the performance of Al_2_O_3_-*x*wt.%TiO_2_ coating. Stevanović [21] studied the effect of heat treatment at 800 °C on the corrosion performance of Al_2_O_3_-TiO_2_-coated ferritic heat-resistant steel. The corrosion resistance of ferritic heat-resistant stainless steel was significantly improved by the Al_2_O_3_-TiO_2_ coating on the surface. The corrosion resistance of the heat-treated coating was significantly improved in the early stage of corrosion. The oxide formed on the surface of the coating during the heat treatment process has a significant impact on the long-term corrosion resistance. Chen [27] prepared an Al_2_O_3_-TiO_2_-MgO coating on a steel surface by plasma spraying and studied the effect of heat treatment at 1000 °C for 24 h on the hardness of the coating. The interaction between the Al-rich and Ti-rich layers in the heat-treated coating and cracking-healing behavior resulting from heat treatment are the main factors that increase the hardness of the coating. Chen [28] studied the effects of heat treatment at 600–1000 °C on the hardness and fracture toughness of Al_2_O_3_-TiO_2_-MgO coatings. Heat treatment can significantly increase the hardness and fracture toughness of the coating, and heat treatment at 1000 °C has the most significant effect. Zhang [29] studied the effect of heat treatment at 200–600 °C for 2 h on the corrosion resistance of TiN_x_/TiO_y_ coatings on a Q235 surface. The corrosion resistance of the heat-treated coating was significantly improved, and the porosity of the coating was reduced by the oxide formed during the heat treatment. Compared with other heating temperatures, the short-time heat treatment at 300 °C had the most significant effect on improving corrosion resistance.

Based on the current state of research, it can be concluded that almost all atmospheric heat treatments cause oxidation, reducing porosity and bridging cracks to significantly improve corrosion resistance. The aim of this work is to study the effect of vacuum heat treatment on the performance of plasma-sprayed Al_2_O_3_-3wt.%TiO_2_ coating on the surface of Q235 steel. Vacuum heat treatment prevents the participation of oxygen, which shows the effect of heat treatment on the Al_2_O_3_-3wt.%TiO_2_ coating more clearly.

## 2. Materials and Methods

Q235 steel (Institute of Metal Research, Chinese Academy of Sciences, Shenyang, China) was used in this study. Its composition (wt.%) was 0.2C, 0.53Mn, 0.30Si, <0.045P, <0.055S, Fe balance. The surfaces were polished with a series of silicon carbide papers, then washed with distilled water, degreased in acetone and dried. Al_2_O_3_-3%TiO_2_ powder with a purity of 99.9% (referred to as AT3) was selected as the coating material. Plasma-spraying technology was used to prepare the coating on the surface of Q234. The plasma-spraying process parameters are shown in Table 1. A vacuum-heat-treatment furnace with a vacuum degree of 6.67 × 10^−3^ MPa was used to process the sprayed samples, and the vacuum-heat-treatment conditions are shown in Table 2. A scanning electron microscope (Hitachi, Japan, model S-4800II(SEM)) equipped with INCA energy-dispersive X-ray spectroscopy was used to observe the morphology and analyze the chemical composition of coating samples. The CS350 electrochemical workstation produced by Wuhan Koster Instrument Co., Ltd., (Wuhan, China) was used to test the electrochemical corrosion performance of the surface coating of the sample. The corrosion solution was prepared with a mass fraction of 5%NaCl, saturated potassium chloride solution and agar. The sample was soaked for 10 min before the start of the test. Open-circuit potential was scanned for anode polarization at a speed of 0.5 mV/s after the open-circuit potential stabilized. The polarization curve formed by fitting Tafel data was used to analyze the corrosion resistance of the coating samples. After the vacuum heat treatment, Rockwell hardness (HRB) measurement was conducted with a load of 100 kg and a loading time of 10 s. The reported value is the average of 10–20 measurements.

## 3. Results and Analysis

### 3.1. Micromorphology of the Coating Surface

**Figure 1:**
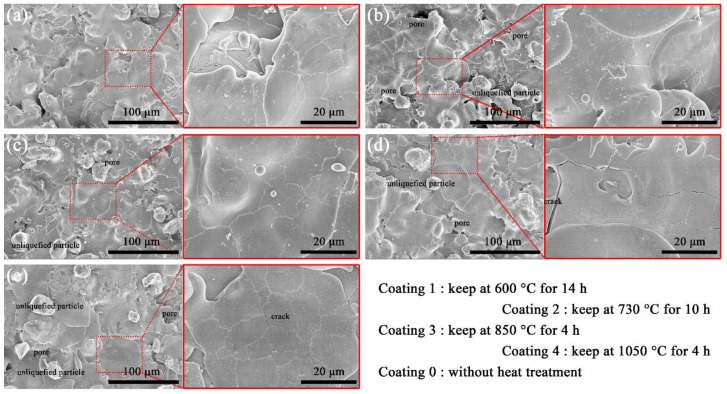
The surface morphology of the coating before electrochemical corrosion: (**a**) coating 1; (**b**) coating 2; (**c**) coating 3; (**d**) coating 4; (**e**) coating 0.

The surface morphology of the coating before electrochemical corrosion is shown in Figure 1. The surfaces of as-sprayed and as-heat-treated coatings were observed with SEM, and they both have a typical lamellar structure, accompanied by a small amount of un-liquefied particles, cracks and pores. adhesion of spherical particles with a diameter of about 30 μm on the surface can be found by observing the boundary. The granular coating material was liquefied by the ultra-high-temperature, forming droplets to cover the surface of Q235. The spherical particles observed on the coating surface were the coating raw materials that were not completely liquefied. The completely liquefied raw-material particles were attached to the surface of the substrate in the form of flakes. AT3 powder was used as the coating material. The liquefied AT3 cracked under the high-temperature gradient in a rapid-solidification process. Pores easily form around non-liquefied particles, which can be observed on the surface of the coating.

Non-liquefied particles, pores and cracks on the coating surface all need to be avoided as much as possible. Compared to the coating without heat treatment, the number of non-liquefied particles, surface pores and cracks in the vacuum-heat-treated coating was slightly lower. The surface of coating 1 is flatter due to the reduction in the number of non-liquefied particles. At present, almost all research on the effect of heat treatment on coating properties uses atmospheric heat treatment. Oxygen enters the coating to form oxides that reduce porosity, which is the main factor improving corrosion resistance [26,29]. However the number of oxides in the coatings presented an obvious increase with increasing temperature, and the corrosion resistance of the coatings showed an obvious reduction [25]. The formation of new oxides is suppressed during vacuum heat treatment. The morphology change of non-liquefied particles, pores and cracks depends only on the diffusion behavior of elements within the coating during vacuum heat treatment. Diffusion behavior is limited in the short-time heat-treatment process, and it fails to bridge the pores and cracks (in Figure 1c). The pores and cracks in the coating are the main factors affecting the coating performance. Atmospheric heat treatment enables oxidation in the coating to fill the pores and bridge the cracks [29]. The effect of vacuum heat treatment on pores and cracks is not obvious. On the contrary, the vacuum heat treatment at 1050 °C promoted cracking (Figure 1d).

**Figure 2:**
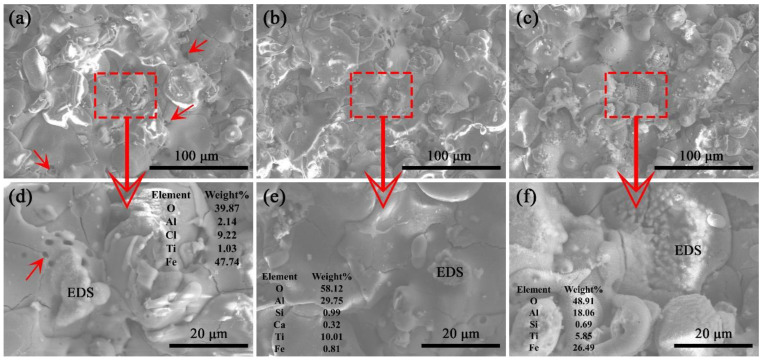
SEM surface morphologies of the coating after electrochemical corrosion: (**a**,**d**) without heat treatment; (**b**,**e**) coating 1; (**c**,**f**) coating 3.

The surface morphology of the coating and EDS results of corrosion products after electrochemical corrosion are shown in Figure 2. Corrosion products were observed on the coating surface. There are many small pores in the unheated coating after corrosion, as indicated by the red arrow in Figure 2. The pores on the coating surface indicate traces of corrosion. The small pores on the coating surface gradually grow up with the progress of corrosion. Compared with the coating that was not heat-treated, the corrosion resistance of the heat-treated coating is better. The main types of corrosion products were analyzed by EDS.

The corrosion products on the surface of the coating in Figure 2 result from the process of electrochemical corrosion. The Fe element in the corrosion product comes from the substrate at the bottom of the coating, which indicates that the corrosion penetrated the entire coating during the electrochemical corrosion process (in Figure 2d). The content of iron oxide in the corrosion products was measured and used to illustrate the corrosion resistance of the coating. The corrosion product on the surface of coating that was not heat-treated is iron oxide. The content of iron oxide in the corrosion products of the coating decreased after heat treatment. The mass fraction of Fe in the corrosion products on the coating surface after vacuum heat treatment was reduced after electrochemical corrosion. The proportion of iron oxides in the corrosion products of coating 1 was the lowest, indicating that the depth of corrosion is the shallowest and the corrosion resistance is better.

### 3.2. Interface Bonding between Coating and Substrate

**Figure 3:**
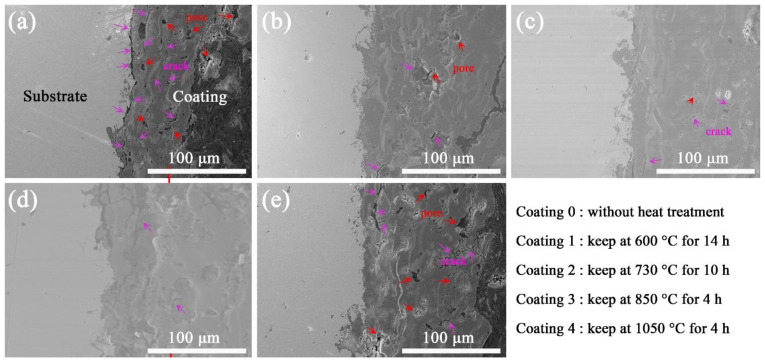
Interface bonding of ceramic coating (**a**) coating 0; (**b**) coating 1; (**c**) coating 2; (**d**) coating 3; (**e**) coating 4.

The interface between the AT3 coating and the substrate was observed by SEM, as shown in Figure 3. More pores and cracks were observed at the interface joint of coating 0 and coating 4 (in Figure 3a). There were few pores and cracks at the interface junction of coatings 1–3, and the coating was tightly combined with the substrate. The liquefied particles were attached to the substrate, forming a sheet-like solidification. The rapid solidification did not provide sufficient time for the diffusion of elements between the coating and the substrate. The physical adsorption without diffusion became the main form of bonding between the coating and the substrate, which is proven by the large number of cracks at the interface junction (in Figure 3a). The tightness of the interface bond between the coating and the substrate was significantly improved after heat treatment. After heat treatment, the coating and the boundary of the substrate were tightly bonded. It was discovered that mechanical bonding can be transformed into metallurgical bonding by inter-diffusion, which is affected by heat treatment [27]. The porosity in the coating is reduced because the atmospheric heat treatment promotes the formation of more oxides and expands the coating [28]. The number of pores and cracks in the coating was also significantly reduced, which is confirmed vacuum-heat-treatment experiments without the participation of additional oxygen. As the heat treatment temperature increases to 1050 °C, diffusion the coating to the substrate increases. A decrease in coating volume is the main reason for the re-formation of pores and cracks within (in Figure 3e). Liu’s research [25] shows that the number of cracks in coatings obviously increases when the temperature of atmospheric heat treatment is increased from 350 °C to 650 °C. In this study, the phenomenon of increased cracks in the coating occurred when the heating temperature reached 1050 °C. Different from the cracking mechanism of the expansion of coating volume caused by the formation of oxides in atmospheric heat treatment, the cracking of the coating in vacuum heat treatment is caused by the different expansion coefficients of Al_2_O_3_ and TiO_2_ [30].

### 3.3. Surface Hardness and Corrosion Resistance

**Figure 4:**
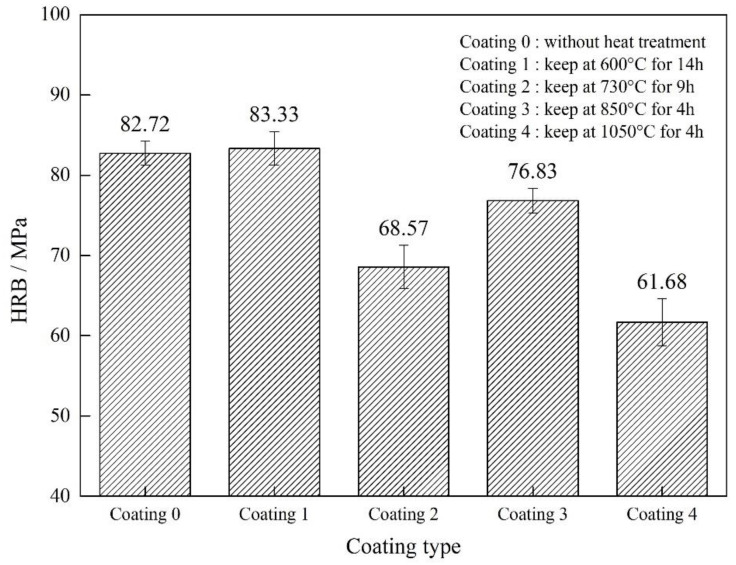
The surface-hardness results of the coatings after different heat treatments.

Figure 4 shows the surface hardness of the coatings after different heat treatments. The hardness of all heat-treated coatings, except for coating 1, was reduced compared with the unheated coating. Both the coating and the metal substrate were affected during the vacuum heat treatment. The coated sample was taken out from the treatment furnace after the temperature was slowly reduced. Significant softening of the metal substrate after heat treatment can be considered, but the results of coating 1 and coating 3 were unexpected. The coating surface and interface of coatings 1 and coating 3 were denser than those of other coatings. The hardness of coatings 1 and 3 were significantly higher than that of the others. The heat-treatment temperature of coating 3 was significantly higher than that of coating 1, which also has a significant softening effect on the substrate. Similarly, the substrate of coating 1 after heat treatment was much softer than that of the unheated coating (equivalent to annealing heat treatment). The overall hardness of the coating 1 sample was slightly higher than that of the unheated sample, which indicates that the hardness of the coating 1 increased after heat treatment.

**Figure 5:**
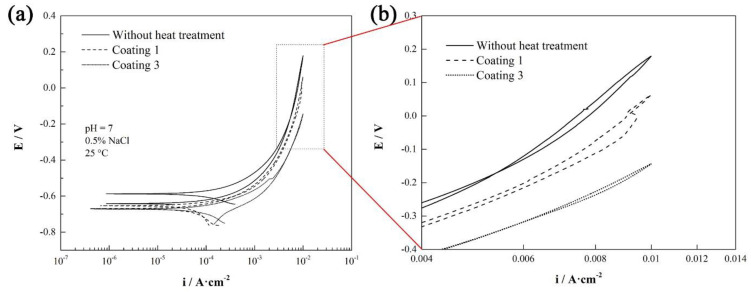
Potentiodynamic polarization curves: (**a**) polarization curve; (**b**) partial enlargement of polarization curve.

The potentiodynamic polarization curves of the different coatings are shown in Figure 5. The result shows that the corrosion-potential Ecorr of coatings were slightly lower than that of the coating without heat treatment. Similarly, the corrosion-current-density Icorr of the coatings with heat treatment was lower, and its corrosion resistance was better, as shown in Table 3. Usually, Icorr is cited as a criterion to evaluate the kinetics of a corrosion process, and it is normally proportional to the corrosion rate [23]. The corrosion-current density of the coating with heat treatment was nearly two-thirds of that of the coating without heat treatment. Similarly, the corrosion rate of the coating with heat treatment was much slower than that of the coating without heat treatment.

Coating 1 represents the coatings after long-term holding at a lower temperature, while coating 3 represents the coatings after short-term holding at a higher temperature. It can be easily observed that the corrosion-current density, corrosion potential, and corrosion rate of coating 1 were 1.997 × 10^−4^ A/cm^2^, −0.47 V and 2.3491 mm/a, respectively, and these results are slightly better than those of coating 3. Thus, the results indicate that coatings heat-treated for a long time at 600 °C have much better corrosion resistance than coatings heat-treated for a short time at 850 °C.

### 3.4. Interface-Element Distribution

**Figure 6:**
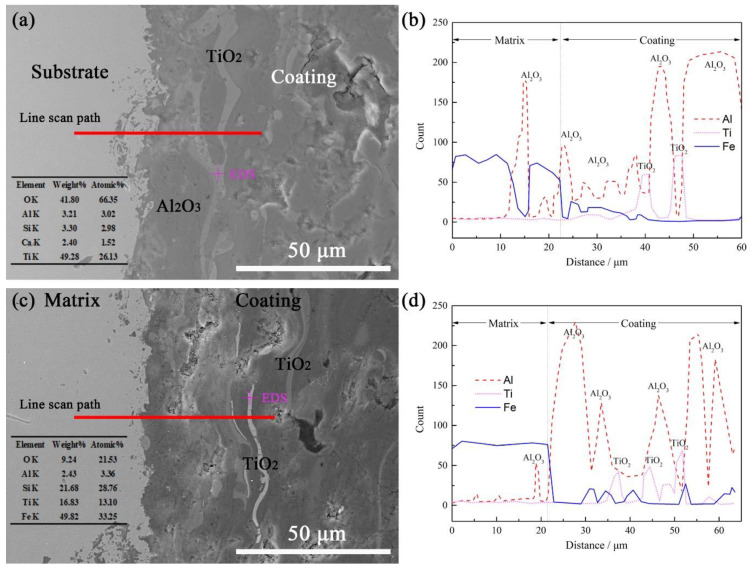
Element distribution between substrate and coating after heat treatment. (**a**) Interface of coating 1; (**b**) line scan of Figure a; (**c**) interface of coating 4; (**d**) line scan of Figure c.

Element distributions between substrate and coating after heat treatment are shown in Figure 6. Coating 1 was kept at 600 °C for 14 h; the dark gray is the main component of Al_2_O_3_, and the small amount of light gray is the TiO_2_ distributed in a curved band. The interface between the substrate and the coating is uneven and rough. This interface is caused by sandblasting before spraying. The uneven surface of the substrate is more conducive to the adhesion of the coating. AT3 powder with a purity of 99.9% was selected as the coating material. After 3%TiO_2_ is liquefied, it infiltrates into the Al_2_O_3_ coating and solidifies, forming the banded segregation. Coating 4 was kept at 1050 °C for 4 h; the dark gray is the main component of Al_2_O_3_, and the small amount of light gray is the TiO_2_ distributed in a curved band. Compared with the coating kept at 600 °C, there is a small amount of Fe infiltrating in the coating after heat-treatment at 1050 °C. Al_2_O_3_ and TiO_2_, as stable oxides, are difficult to decompose at high temperatures, and Fe in the substrate diffuses into the layer in the heat treatment at 1050 °C (in Figure 6d).

A metallurgical bond was formed, which can be judged from the distribution of Al and Fe elements. This result is consistent with the previous studies [28]. However, the vacuum heat treatment promotes the diffusion of Fe in atom form instead of oxide form, and this diffusion could facilitate the combination of the substrate and the coating.

**Figure 7:**
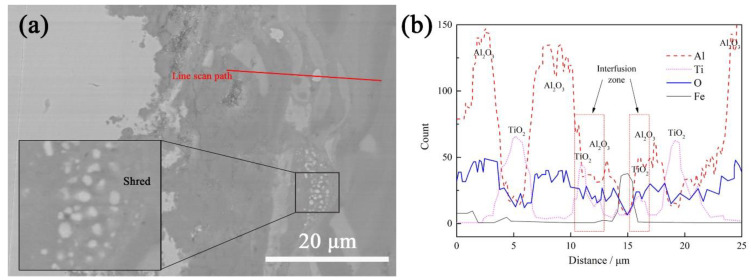
Diffusion of elements in the coating after heat treatment. (**a**) Cross-sectional morphology of the coating after heat treatment; (**b**) line scan.

The element diffusions in the coating after heat treatment are shown in Figure 7. White granular iron, light gray rod-shaped TiO_2_ and continuous dark gray Al_2_O_3_ are easily observed in the coating (in Figure 7a). The inter-diffusion zone of Al_2_O_3_ and TiO_2_ can be found by line scanning, and these two components are fused with one another. It has been reported [27] that one of the reasons why the heat treatment can increase the surface hardness of the coating is the formation of an Al-rich and Ti-rich diffusion layer. This indicates that the inter-diffusion layer formed by Al_2_O_3_ and TiO_2_ is the main reason improving the hardness and corrosion resistance of the AT3 coating. The vacuum heat treatment eliminates the influence of newly formed oxides on coating performance. According to Figure 6 and Figure 7, the effect of heat treatment on the diffusion of elements in the coating is greater than that in the substrate. Due to the lack of oxygen in the vacuum heat treatment, no new oxides are formed. The iron in the substrate can enter the coating in elemental form and be observed after heat treatment at 1050 °C. Vacuum heat treatment can not only promote the mutual fusion of Al_2_O_3_ and TiO_2_ but also fragment the phases in the coating.

The higher the temperature, the greater the impact on the coating, while the softening effect on the substrate also increases. As the heating temperature and the holding time increase, the increase in the hardness of the coating is insufficient to compensate for the significant decrease in the hardness of the substrate. As the heating temperature increases and the holding time increases, the increase in the hardness of the coating is insufficient to compensate for the significant decrease in the hardness of the substrate. Similarly, the heat-treated coating can improve the corrosion resistance, according to the results in Table 3. The fusion of Al_2_O_3_ and TiO_2_ and the fragmentation of phases in the coating reduce the electrode potential in the coating. Unlike ordinary heat treatment, which involves the participation of oxygen, vacuum heat treatment does not form additional oxides. The formation of new oxides in the coating reduces the porosity and is the main reason for the improved corrosion resistance after atmospheric heat treatment. The uniformity of TiO_2_ distribution inside alumina is improved, and the bonding tightness between the coating and the substrate is also improved, which is the main factor improving corrosion resistance during vacuum heat treatment.

## 4. Conclusions

By observing the surface and interface of the coating and testing the surface hardness and corrosion resistance, the effect of vacuum heat treatment on coatings was studied, and the results of observation and analysis are as follows:

(1) By comparing the surface morphology of the coating, new oxide was observed to be produced after vacuum heat treatment. The corrosion products on the coating surface after vacuum heat treatment have less Fe content, and heat treatment effectively hinders corrosion depth.
(2) Vacuum heat treatment can strengthen the interface between the substrate and the coating, and the interface is more closely bonded. The effect of vacuum heat treatment on coating performance is mainly caused by the mutual fusion of Al_2_O_3_ and TiO_2_ in the coating and the fragmentation of the phase in the coating. Vacuum heat treatment improves the surface hardness and corrosion resistance.
(3) Coating performance is improved, while the Q235 performance decreases with an increase in heating temperature and holding time in vacuum heat treatment. Compared with other parameters, the vacuum heat treatment at 600 °C for 14 h can simultaneously improve the surface hardness of AT3 coatings and the corrosion resistance of the coating sample.

## Figures and Tables

**Table 1:** Plasma-spraying process parameters.

Voltage/V	Elective Current/A	Argon Pressure/Mpa	Spray Distance/mm	Argon Pressure/(kg·cm^−2^)	Powder Delivery/(L·h^−1^)	Powderfeeding Voltage/V
27	580	0.7	100	100	300	3

**Table 2:** Vacuum-heat-treatment process parameters for coated samples.

Sample Name	Heating Temperature/°C	Holding Time/h
Coating 0	Without heat treatment	
Coating 1	600	14
Coating 2	730	10
Coating 3	850	4
Coating 4	1050	4

**Table 3:** Tafel data-fitting table.

Samples	Ecorr (Volts)	Icorr (A/cm^2^)	Corrosion Rate (mm/a)
coating 0	−0.58737	3.246 × 10^−4^	3.8174
coating 1	−0.66956	1.997 × 10^−4^	2.3491
coating 3	−0.67271	2.331 × 10^−4^	2.6239
